# Lack of IL-6 during Coxsackievirus Infection Heightens the Early Immune Response Resulting in Increased Severity of Chronic Autoimmune Myocarditis

**DOI:** 10.1371/journal.pone.0006207

**Published:** 2009-07-09

**Authors:** Maya C. Poffenberger, Nadine Straka, Nahida El Warry, Dianne Fang, Iryna Shanina, Marc S. Horwitz

**Affiliations:** Microbiology and Immunology, The University of British Columbia, Vancouver, British Columbia, Canada; New York University School of Medicine, United States of America

## Abstract

**Background:**

Chronic myocarditis is often initiated by viral infection, the most common of which is coxsackievirus infection. The precise mechanism by which viral infection leads to chronic autoimmune pathology is poorly understood, however it is clear that the early immune response plays a critical role. Previous results have shown that the inflammatory cytokine interleukin (IL)-6 is integral to the development of experimental-induced autoimmune myocarditis. However, the function of IL-6 during viral-mediated autoimmunity has yet to be elucidated.

**Methods and Results:**

To address the requirement of IL-6 during disease induction, IL-6 deficient mice were infected with coxsackievirus B3 (CB3). Following infection, mice lacking IL-6 developed increased chronic autoimmune disease pathology compared to wild type controls without a corresponding change in the level of viral replication in the heart. This increase in disease severity was accompanied by elevated levels of TNF-α, MCP-1, IL-10, activated T cells and cardiac infiltrating macrophage/monocytes. Injection of recombinant IL-6 early following infection in the IL-6 deficient mice was sufficient to lower the serum cytokines TNF-α and IL-10 as well as the serum chemokines MCP-1, MIP-1β, RANTES and MIG with a corresponding decrease in the chronic disease pathology strongly suggests an important regulatory role for IL-6 during the early response.

**Conclusions:**

While IL-6 plays a pathogenic role in experimental-induced autoimmune disease, its function following viral-induced autoimmunity is not reprised. By regulating the early immune response and thereby controlling the severity of chronic disease, IL-6 directs the outcome of chronic autoimmune myocarditis.

## Introduction

Autoimmune myocarditis, a precursor stage of dilated cardiomyopathy (DCM), is the leading cause of sudden death in young adults [1]. Disease progression to DCM leaves heart transplantation as the only treatment option. DCM is often caused by enterovirus infection, predominantly the cardiotropic virus coxsackievirus [1]. Following coxsackievirus B3 (CB3) infection, an acute disease stage ensues in which heart damage is viral-mediated. In genetically susceptible individuals, disease can progress to a chronic autoimmune phase characterized by infiltration of autoreactive lymphocytes and fibrosis of the heart tissue. While the mechanism by which viral infection leads to autoimmune myocarditis is not completely understood, there is compelling evidence to suggest that interleukin (IL)-6 is involved [2], [3], [4], [5].

IL-6 is a pleotropic cytokine that acts during both pro- and anti-inflammatory responses and has been observed in the context of infection, inflammation and autoimmunity. IL-6 is typically induced following pathogen stimulation as a part of the innate inflammatory response and its major functions include initiation of the acute-phase response, activation of T cells and stimulation of B cell immunoglobulin production [6]. More recently, IL-6 has been linked to the release of otherwise suppressed effector T cells [7], inhibition of TGF-β induced regulatory T (Treg) cell generation [8] and induction of Th17 cells as a co-factor with TGF-β [9]. Further, signaling through gp130, a component of the IL-6 receptor complex, protects cardiac myocytes from CB3 infection directly [10]. IL-6 functions globally in response to inflammation and infection and it is important in our understanding of the development of autoimmunity.

Specifically, lack of IL-6 has been demonstrated to be sufficient to prevent a plethora of autoimmune diseases such as experimental autoimmune encephalomyelitis (EAE) [11], pristane-induced lupus [12], experimental-induced arthritis [13], [14], autoimmune myasthenia gravis [15] and, most notably with regard to this report, experimental-induced autoimmune myocarditis (EAM) [2]. Following injection of cardiac myosin peptide emulsified in complete Freund's adjuvant (CFA), EAM did not develop in IL-6 deficient mice (IL-6KO, BALB/c) [2]. IL-6KO mice had decreased levels of TNF-α, complement component 3 and co-stimulatory molecules following induction of disease. While EAM is a useful model of myocarditis that allows for study of the chronic stage of disease without the complication of the viral infection, it does not mimic the natural acquisition of disease and lacks the complexity of the response to virus. Thus, it is valuable to decipher the role of IL-6 in the context of viral-induced autoimmune myocarditis.

In this report, we demonstrate that IL-6 regulates the development of CB3-mediated chronic myocarditis. In contrast to the EAM findings, IL-6KO mice infected with CB3 developed significantly greater chronic heart disease. Heightened disease was associated with increases in early inflammatory responses including serum levels of TNF-α, IL-10 and MCP-1 as well as T cell activation and cardiac monocyte/macrophage infiltration. The early inflammatory response and chronic disease severity was controlled by supplementing the viral infection with recombinant IL-6 indicating that IL-6 functions as a regulator of the early host response and has a protective role during the progression of CB3-induced chronic myocarditis.

## Materials and Methods

### Animals

IL-6 deficient C57BL/6 (IL-6KO) and C57BL/6 mice were purchased from The Jackson Laboratory (Bar Harbor, ME). These mice were maintained in the Wesbrook Animal Facility at the University of British Columbia (UBC).

### Virus

Coxsackievirus group B type 3 (CB3) (Nancy strain) was obtained from Dr. J.K. Chantler (UBC). Virus stocks of CB3 were prepared as previously described [16]. Virus stocks were stored at −80°C. Mice 8–12 weeks of age were infected intraperitoneally with a sublethal dose of 100 PFU of CB3 in Dulbecco's Modified Eagle Medium (DMEM) for the initial experiments. Subsequently, mice were infected with 10,000 PFU of CB3 in DMEM and this includes the rIL-6 addition experiments.

The IL-6KO mice are only commercially available on the C57BL/6 background which is genetically resistant to CB3-induced chronic autoimmune myocarditis, therefore, co-treatment with LPS at the time of infection is required to overcome genetic resistance [17]. C57BL/6 and IL-6KO mice were treated with CB3, CB3 plus 25 µg LPS (*S. minnesota*) (CB3/LPS), 25 µg LPS (*S. minnesota*) (Sigma-Aldrich, St. Louis, MO) or DMEM. Mice were sacrificed at day 10 (acute phase) or day 28 (chronic phase) post infection. Where noted, mice were injected with 50 ng/mouse recombinant mouse IL-6 (rIL-6) (eBioscience, San Diego, CA) or DMEM at days 0, 1, 2 and 3 post treatment with CB3/LPS.

### Quantitation of the replicative virus in the heart

Viral load was quantitated by plaque assay at day 5 post infection. Heart samples were collected, weighed and homogenized at 10% weight/volume in DMEM. Dilutions of homogenates were incubated at 37°C with rocking for one hour on a confluent monolayer of HeLa cells (ATCC CCL-2). Plates were incubated for three days to allow plaque formation. Plaque assays were performed in duplicate.

### Histology

At sacrifice, heart tissue was removed for paraffin sectioning. Tissue was processed and stained by Wax-it Histology Services Inc. (UBC). Sections of 4 micron thickness were cut for staining. Tissue was stained by standard protocols with hematoxylin and eosin as well as Masson's trichrome to detect damage by cellular infiltration and fibrosis. Serial sections of the heart were scored blindly according to a 4-tier scoring system: grade 1, 0–10% pathology; grade 2, 11–25%; grade 3, 26–50%; grade 4, greater than 50%.

### Flow cytometry

At sacrifice, spleen tissue was removed and placed in phosphate buffered saline (PBS) buffer on ice. Single cell suspensions of splenocytes were isolated by standard procedures. Cells were treated with Mouse BD FcBlock™ (clone 2.4G2) (BD Pharmingen, San Jose, CA) for 15 minutes prior to staining. Flow cytometry was performed on a FACS Calibur or a LSRII (BD Biosciences, San Jose, CA) and analysed with FlowJo (Tree Star Inc., Ashland, Oregon) software.

### Antibodies

The following antibodies were purchased from BD Pharmingen: biotin-anti-CD69 (clone H1.2F3), biotin-anti-CD44 (clone IM7), biotin-anti-CD49b (clone DX5), biotin-anti-CD3 (clone 145-2C11), biotin-anti-CD62L (clone MEL-14), APC-anti-CD11b (clone M1/70), biotin-anti-CD80 (clone 16-10A1), biotin-anti-CD86 (clone GL1) and biotin-anti-CD40 (clone 3/23). The following antibodies were purchased from eBioscience: APC-anti-CD4 (clone RM4-5), FITC-anti-CD8 (clone 53–6.2), FITC-anti-Foxp3 (clone FJK-16s), PE-anti-RORγt (clone AFKJS-9), PE-anti-CD25 (clone PC61), FITC-anti-CD11c (clone N418), biotin-anti-CD14 (clone Sa2-8) and biotin-anti-F4/80 (clone BM8). Hamster IgG1λ (BD Pharmingen), rat IgG2aΚ (BD Pharmingen) and rat IgG2a (eBioscience) were used as isotype controls for CD69, Foxp3 and RORγt staining respectively. Streptavidin-PE (BD Pharmingen) or Streptavidin-PECy-7 (eBioscience) were used as secondary antibody to visualize biotinylated antibodies.

### Cytometric bead array

Serum was collected at days 1, 3, 7 and14 post treatments with DMEM, LPS, CB3 or CB3/LPS and stored at −80°C until analysis. Concentrations of TNF-α, IFN-γ, IL-6, IL-12p70, IL-10 and MCP-1 were determined with a BD Cytometric Bead Array (CBA) Mouse Inflammation kit according to manufacturers' instructions (BD Biosciences). Concentrations of MIP-1α, MIP-1β, MIG and RANTES were determined from serum samples collected at day 3 post CB3/LPS treatment with rIL-6 or DMEM treatment at days 0, 1 and 2 PI using a BD CBA flex set kit according to manufacturers' instructions (BD Biosciences). Data was analyzed with FCAP Array software (BD Biosciences).

### Isolation of heart infiltrate

Mice were anesthetized with 3.2 mg Ketalean (Bimeda-MTC, Dublin, Ireland) and 0.08 mg Xylazine (Bayer, Leverkusen, Germany) and perfused with sterile PBS at 7 or 10 days post CB3/LPS treatment. Hearts were minced and digested with 0.2% collagenase (Sigma-Aldrich), 0.25% pancreatin (Sigma-Aldrich) and 0.1% DNase (Roche Applied Science, Basel, Switzerland) for 7 minutes at 37°C. Digestion was stopped with addition of 0.1M EDTA. Following digestion, single cell suspensions of heart infiltrate were isolated by standard procedures and cells were stained for flow cytometry.

### Statistics

Statistical analysis was done by unpaired Student's t-test for all plaque assay, cytokine analysis and immune cell population analysis. P values of <0.05 (*) were considered significant. Mann Whitney U test was used to calculate significance for differences in disease severity.

## Results

### Increased severity of chronic myocarditis in IL-6KO mice

**Figure 1:**
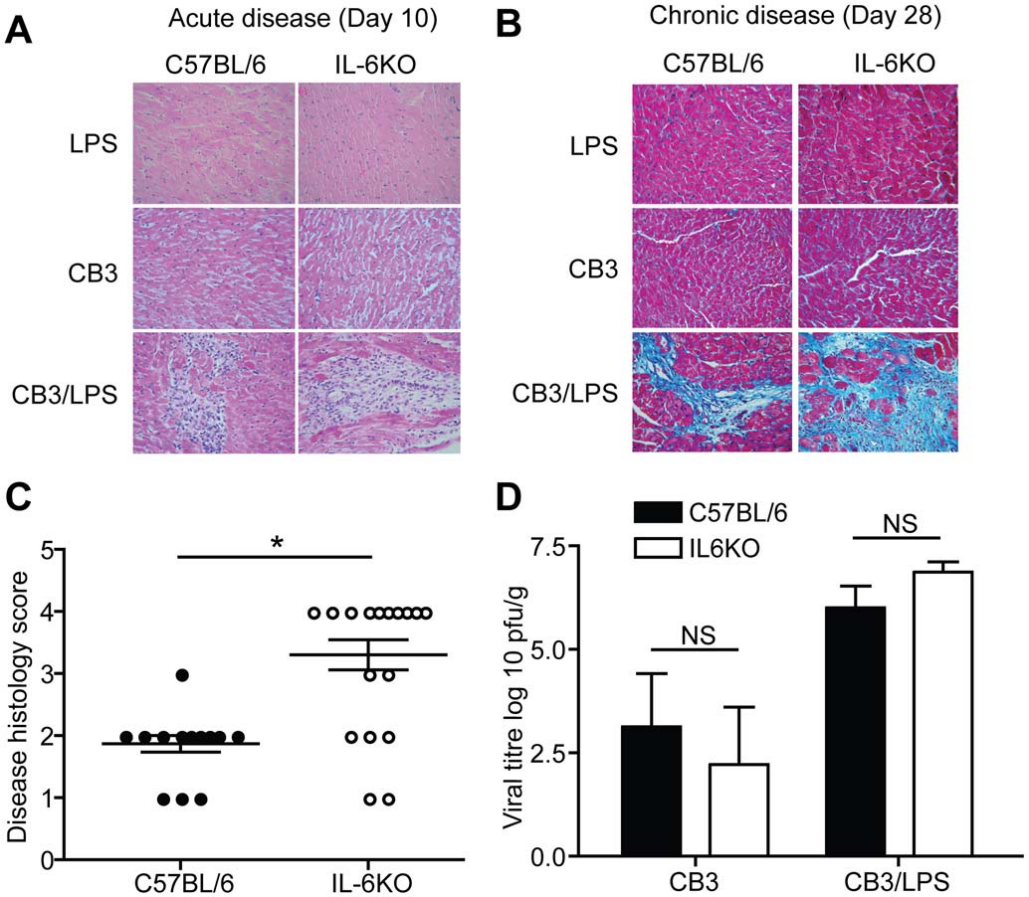
IL-6KO mice develop increased chronic myocarditis severity without an increase in virus in the heart. (A) Representative Hematoxylin and Eosin stained cardiac sections from C57BL/6 or IL-6KO mice. Mice treated with LPS or CB3 alone did not develop acute disease lesions at day 10 PI (LPS: C57BL/6 n = 4, IL-6KO n = 4) (CB3: C57BL/6 n = 5, IL-6KO n = 4). Mice treated with CB3/LPS developed lesions and immune cell infiltration at 10 days PI (C57BL/6 n = 4, IL-6KO n = 4). Magnification:400×. (B) Representative Masson's Trichrome stained cardiac section from C57BL/6 or IL-6KO mice. Following treatment with CB3/LPS, mice developed disease pathology as determined by fibrosis in blue and immune cell infiltration within the fibrosis areas by 28 days PI (C57BL/6 n = 15, IL-6KO n = 20). Mice infected with LPS or CB3 alone did not develop significant chronic disease pathology (LPS: C57BL/6 n = 5, IL-6KO n = 5) (CB3: C57BL/6 n = 6, IL-6KO n = 7). Magnification:400×. (C) Chronic cardiac disease histology was scored blindly by a four tier grading system to determine severity differences: grade 1, 0–10% pathology; grade 2, 11–25%; grade 3, 26–50%; grade 4, greater than 50% (black circles indicate wt mice, white circles indicate IL-6KO mice) (bar is mean±SE, *p<0.05). Disease severity was found to be significantly higher in the IL-6KO mice compared to wild type controls. (D) Quantification of replicative virus in the heart post infection showed no significant differences in viral titer between IL-6KO and wt mice at day 5 post infection (CB3: C57BL/6 n = 5, IL-6KO n = 5) (CB3/LPS: C57BL/6 n = 5, IL-6KO n = 5) (black bars indicate wt mice, white bars indicate IL-6KO mice) (mean±SE, NS = not significant). This indicates that IL-6 is not essential for control of viral replication in the heart.

To determine the role of IL-6 in the viral induction of autoimmune myocarditis, myocarditis was induced in IL-6KO and wild type C57BL/6 (wt) mice by injection of CB3 plus LPS (*S. minnesota*) (CB3/LPS). Control mice were concurrently challenged with CB3, LPS (*S. minnesota*) alone or DMEM. Heart tissue from mice sacrificed at day 10 PI showed pathology characteristic of the acute phase of myocarditis in both C57BL/6 and IL-6KO mice treated with CB3/LPS (Figure 1a). Acute disease pathology was not observed in IL-6KO mice treated with LPS or CB3 alone indicating that, as with wt mice, disruption of IL-6 does not alter the general immune response enough to allow LPS or CB3 alone to cause disease (Figure 1a). Heart tissue harvested from mice at day 28 PI showed fibrosis and immune cell infiltration characteristic of chronic myocarditis in both IL-6KO and wt mice treated with CB3/LPS (Figure 1b) indicating the IL-6 is not required for chronic myocarditis development. This chronic disease was autoimmune in nature as transfer of splenocytes from CB3/LPS treated IL-6KO mice into naïve RAG deficient mice was sufficient to induce disease (Data not shown). As expected, mice treated with CB3, LPS or DMEM alone showed no signs of chronic myocarditis or fibrosis at day 28 (Figure 1b, Data not shown).

Comparison of chronic disease severity between the IL-6KO mice and wt controls showed significantly greater disease pathology in IL-6KO mice as compared to wt mice (Figure 1c). Over 65% of IL-6KO mice presented with infiltrating lesions and fibrosis with grade 4 severity, while 93% of wt mice had grade 2 or less heart pathology. Therefore, lack of IL-6 results in greater chronic disease severity post-infection and is indicative of a regulatory role for IL-6 in the host response to infection and the ensuing chronic pathology.

### Cardiac viral replication in IL-6KO mice is similar to wt controls

The increased cardiac disease pathology observed in IL-6KO mice could be a direct result of the inability to control viral infection in the heart. To determine whether differences in viral numbers were responsible for the increased pathology, quantitative viral plaque assays were performed. No significant difference in the replicating viral numbers in the heart was observed between IL-6KO and wt mice at day 5 PI (Figure 1d). This suggests that the differences in disease severity were not a direct result of an inability of IL-6KO mice to control cardiac infection.

### Increased CD69 expression by T cells in CB3-infected IL-6KO but not in wt mice

**Figure 2:**
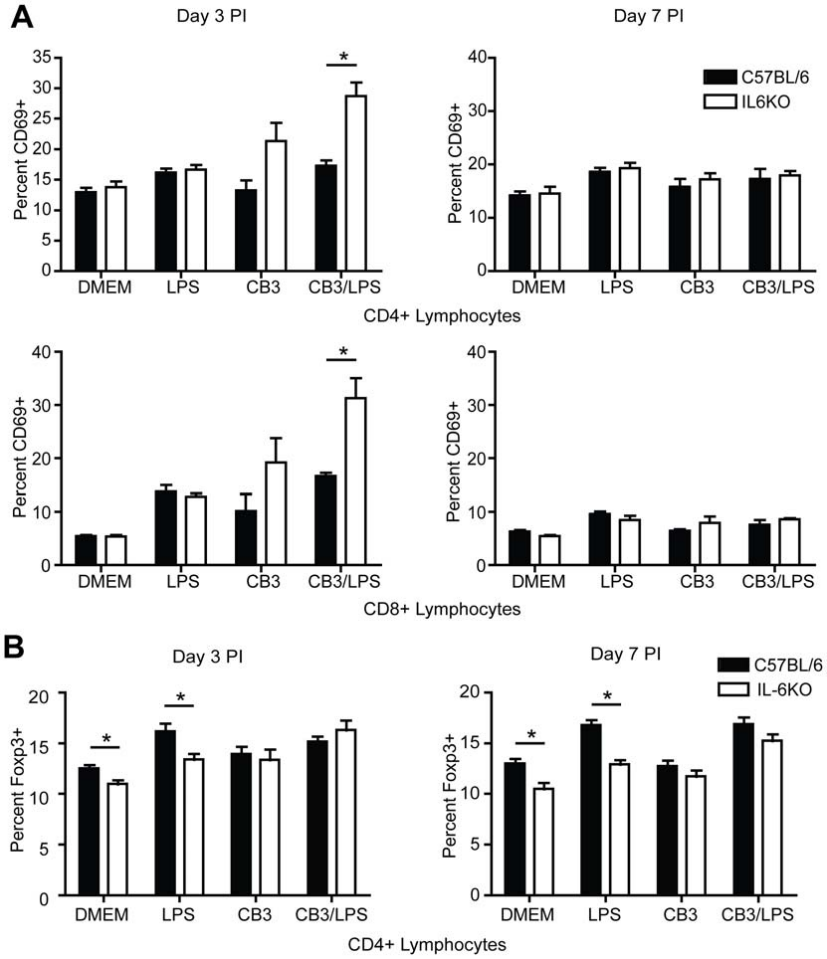
IL-6KO mice have increased expression of the early activation marker CD69 at 3 days PI. (A) T cell activation was monitored by flow cytometry analysis of splenocytes following DMEM, LPS, CB3 or CB3/LPS treatment. Activated T cells were identified by the surface markers CD4, CD8 and CD69. CD69 expression was significantly increased on both CD4+ and CD8+ T lymphocytes at 3 days post infection (DMEM: C57BL/6 n = 4, IL-6KO n = 4) (LPS: C57BL/6 n = 7, IL-6KO n = 8) (CB3: C57BL/6 n = 8, IL-6KO n = 18) (CB3/LPS: C57BL/6 n = 8, IL-6KO n = 17) (black bars indicate wt mice, white bars indicate IL-6KO mice) (mean±SE, *p<0.05) and abrogated by 7 days post infection (DMEM: C57BL/6 n = 4, IL-6KO n = 4) (LPS: C57BL/6 n = 4, IL-6KO n = 4) (CB3: C57BL/6 n = 4, IL-6KO n = 4) (CB3/LPS: C57BL/6 n = 4, IL-6KO n = 5). (B) The percent of CD4+ Treg cells was determined by FACS of splenocytes at 3 and 7 days post DMEM, LPS, CB3 or CB3/LPS treatment. Treg cells were identified by expression of CD4 and the transcription factor Foxp3. IL-6KO mice with DMEM treatment contained a significantly lower percentage of CD4+ Foxp3+ cells compared to the wild type controls with DMEM treatment (DMEM: C57BL/6 n = 8 at day 3 n = 7 at day 7, IL-6KO n = 8 at day 3 n = 7 at day 7) (black bars indicate wt mice, white bars indicate IL-6KO mice) (mean±SE, *p<0.05). However, following treatment with CB3 or CB3/LPS but not LPS alone, the percentage of CD4+ Foxp3+ cells was not significantly different (LPS: C57BL/6 n = 8 at day 3 n = 12 at day 7, IL-6KO n = 8 at day 3 n = 14 at day 7) (CB3: C57BL/6 n = 8 at day 3 n = 11 at day 7, IL-6KO n = 8 at day 3 n = 17 at day 7) (CB3/LPS: C57BL/6 n = 9 at day 3 n = 13 at day 7, IL-6KO n = 9 at day 3 n = 18 at day 7) (mean±SE, *p<0.05) suggesting sufficient proportions of Treg cells are present to immunosuppress disease.

To examine whether changes in the antigen presenting cells (APC) or the T cell response following infection were responsible for the increase in disease severity, an analysis of immune cells post-infection was performed. No differences were observed in the percentages of immune cell populations present as measured by APC markers CD11b and CD11c or the T cell co-receptors CD4 and CD8. Within these cell populations, no differences in activation state were observed as measured by the APC co-stimulatory markers CD80, CD86 or CD40 or the T cell activation markers CD44, CD62L or CD25 (Data not shown). However, in IL-6KO mice, the T cell activation marker, CD69, was significantly upregulated on both CD4+ and CD8+ T cells at day 3 PI as compared to wt controls (Figure 2a, Figure S1). By day 7 however, this difference in CD69 expression had diminished and was similar to that found on T cells in wt mice (Figure 2a, Figure S1). As CD69 is the only activation marker to be upregulated in the IL6KO mice, the increase in CD69+ cells reflects an important early T cell regulatory role for IL-6 and these cells may be integral to the increase in disease severity.

Based on data suggesting that IL-6 is important in Treg induction and function [7], [8], we monitored changes in Treg cell populations over the course of infection. Tregs were identified using the cell markers CD4, CD25 and the Treg specific transcription factor, Foxp3. While the steady state ratio of Treg cells to CD4+ lymphocytes was found to be significantly lower in the IL-6KO mice compared to wt mice, following disease induction the ratio of Treg cells to CD4+ T cells did not significantly differ (Figure 2b, Figure S2). This suggests that there is likely a sufficient proportion of Treg cells present to regulate the T cell response following infection and we found no evidence to suggest that lack of IL-6 resulted in changes in the Treg population that influenced the severity of pathology observed post-infection in the heart.

### Early upregulation of MCP-1, TNF-α and IL-10 in IL-6KO mice

**Figure 3:**
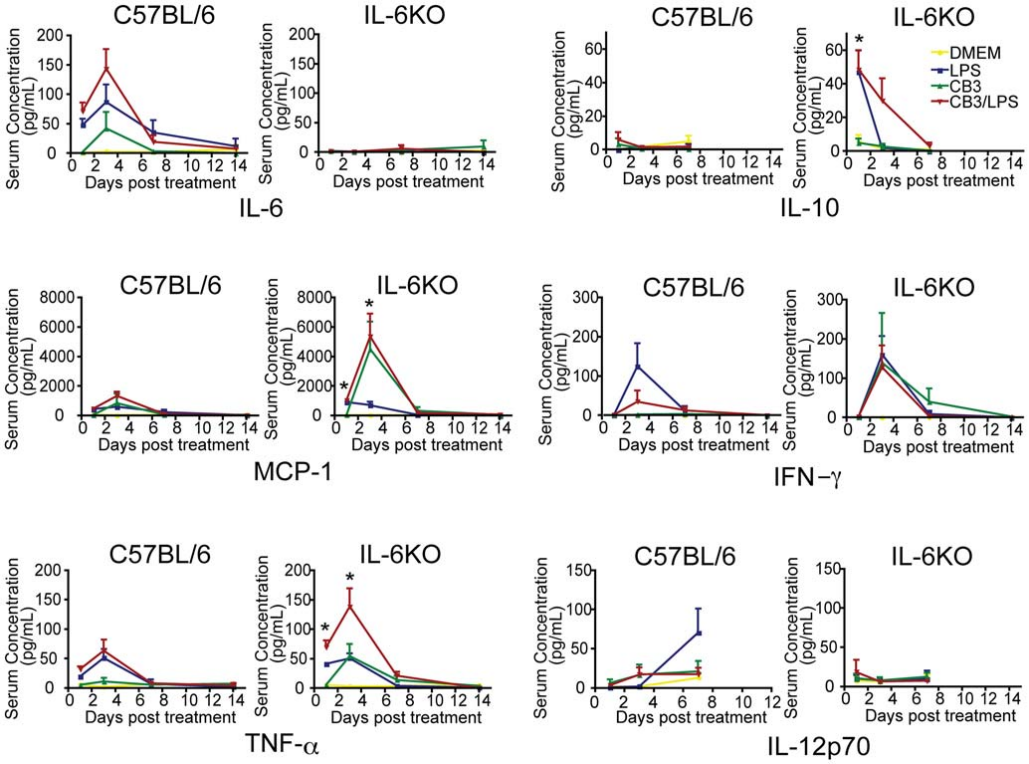
TNF-α, IL-10 and MCP-1 expression is increased in the absence of IL-6. The serum concentrations of the cytokines IL-12p70, IL-6, IL-10, IFNγ, TNF-α and the chemokine MCP-1 were monitored following treatment with DMEM, LPS, CB3 and CB3/LPS by a BD cytometric bead array inflammation assay (n≥3 for each treatment group at day 1, 3 and 7, n≥2 for each treatment group at day 14) (mean±SE, *p<0.05). IL-6 increased following infection with CB3 indicating a role for IL-6 in the response to viral infection. TNF-α, IL-10 and MCP-1 were significantly increased in the IL-6KO mice compared to the wild type controls indicating a possible role for IL-6 in the regulation of these immune components (orange diamonds indicate DMEM treatment, green squares indicate LPS treatment, blue triangles indicate CB3 treatment, red circles indicate CB3/LPS treatment).

To determine if changes in the inflammatory response post-infection are responsible for the differences in disease severity, serum was assayed post infection using the BD CBA kit to measure inflammatory cytokine levels. Serum was collected at 1, 3, 7 and 14 days PI and assayed for TNF-α, IFN-γ, IL-6, IL-12p70, IL-10 and MCP-1 (Figure 3). In response to CB3 infection, wt mice, but not IL-6KO mice, upregulated IL-6 with a peak in production at day 3 post infection indicating that IL-6 is involved in the early immune response following coxsackievirus infection (Figure 3).

IFN-γ and IL-12p70 levels increased at a similar rate in both IL-6KO and wt controls following CB3/LPS treatment (Figure 3). However, the levels of TNF-α, IL-10 and MCP-1 were increased in IL-6KO mice compared to the wt controls (Figure 3). TNF-α upregulation occurred following CB3/LPS and LPS treatment in wt mice and CB3/LPS, CB3 and LPS treatment in the IL-6KO mouse. An additive effect with CB3 and LPS treatment was observed only in the absence of IL-6. Conversely, increased IL-10 production in IL-6KO mice corresponded to the presence of LPS as levels were elevated following LPS or CB3/LPS treatment. This was only observed in the absence of IL-6. This strongly suggests that production of the immunosuppressive cytokine IL-10 is regulated in part by IL-6. The change in MCP-1 levels in IL6KO mice corresponded to viral infection as both CB3 and CB3/LPS treatment resulted in increased production whereas LPS treatment alone did not. This suggests that MCP-1 is regulated by IL-6 following CB3 infection. Taken together, these results suggest that in the absence of IL-6, there is an imbalance in the induction of early inflammatory modulators and that IL-6 likely functions as a regulator of the early immune response to infection.

### Increased cardiac infiltration of macrophage/monocytes in CB3-infected IL-6KO mice

**Figure 4:**
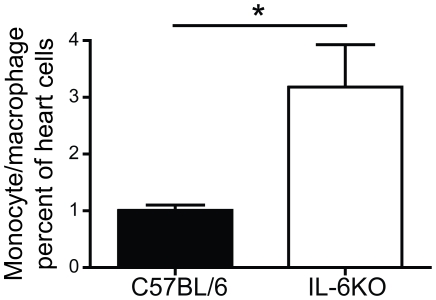
IL-6KO mice have increased monocyte/macrophage infiltration into the heart at 7 days post infection. Heart infiltrate was measured by flow cytometry analysis at 7 days post treatment with CB3/LPS. Cardiac infiltrate determined based on forward and side scatter as well as CD11b and CD11c immunostaining. IL-6KO mice had significantly increased monocyte/macrophage infiltration at 7 days post treatment compared to wt mice (C57BL/6 n = 9, IL-6KO n = 11) (black bars indicate wt mice, white bars indicate IL-6KO mice) (mean±SE, *p<0.05). This increased correlated with the elevated levels of the chemokine MCP-1 in IL-6KO mice and suggests a link between these two factors.

Increased levels of the chemokine MCP-1 have previously been associated with increased migration of mononuclear cells to the heart at 7 days post CB3 infection [18]. To determine if the heightened disease severity and MCP-1 production in IL-6KO mice reflected an increase in mononuclear cell infiltration in the heart during the initiation of disease, flow cytometry was used to directly characterize the immune cells infiltrating the heart (day 7 PI). Significant increases in the infiltration of monocyte/macrophages, as determined by forward and side scatter and based on surface expression of CD11b and CD11c, was observed in the hearts of IL-6KO mice treated with CB3/LPS as compared to their wt counterparts (Figure 4, Figure S3). These CD11b+ cells were further determined to be 81.3%±1.1% F4/80 positive and 59.7%±2.8% CD14 positive identifying them as primarily monocyte/macrophages. No other significant difference in the immune cell infiltration was observed (Data not shown). This demonstrates that greater cardiac infiltration by macrophage/monocytes occurred in association with an increase in chronic disease severity following infection of IL-6KO mice. This strongly suggests that heightened MCP-1 levels in the absence of IL-6 leads to greater immune cell infiltration, specifically CD11b+ cells, and subsequent cardiac pathology.

### Recombinant IL-6 treatment in IL-6KO mice results in decreased chronic disease severity

**Figure 5:**
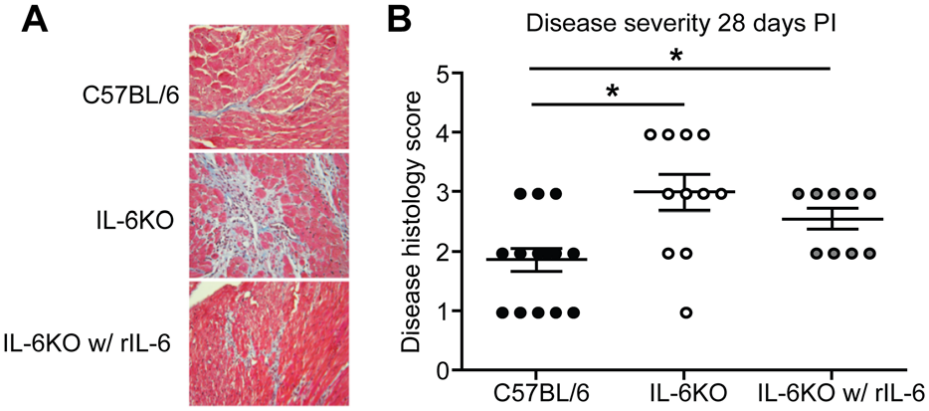
Injection of rIL-6 in IL-6KO mice decreased chronic disease severity following CB3/LPS treatment. (A) Representative Masson's Trichrome stained cardiac section from C57BL/6 or IL-6KO mice. Mice were treated with CB3/LPS and either DMEM or rIL-6 on days 0, 1, 2 and 3 post infection. Mice developed disease pathology as determined by fibrosis in blue and immune cell infiltration within the fibrosis areas by 28 days PI (C57BL/6 with DMEM n = 15, IL-6KO with DMEM n = 11, IL-6KO with rIL-6 n = 9). Magnification:400×. (B) Chronic cardiac disease histology was scored blindly by a four tier grading system to determine severity differences: grade 1, 0–10% pathology; grade 2, 11–25%; grade 3, 26–50%; grade 4, greater than 50% (black circles indicate wt mice with DMEM, white circles indicate IL-6KO mice with DMEM, grey circles indicate IL-6KO mice with rIL-6) (bar is mean±SE, *p<0.05). Disease severity was found to be decreased in the IL-6KO mice treated with rIL-6 compared to DMEM treated IL-6KO mice.

The increased autoimmune disease observed in IL-6KO mice could be due to an early developmental defect in these mice rather than a lack of IL-6 specifically during the early immune response. To determine if IL-6 production during the early immune response regulates the chronic disease severity, recombinant IL-6 (rIL-6) or DMEM was administered to IL-6KO mice at days 0, 1, 2 and 3 PI and the chronic disease severity was observed at 28 days PI. IL-6KO mice were found to have significantly increased chronic disease severity compared to wild type controls, however, treatment with rIL-6 was sufficient to decrease the chronic disease histology (Figure 5a and 5b). This conclusively shows that IL-6 regulates chronic disease severity by regulating the early immune response following viral infection.

### Recombinant IL-6 regulates early inflammatory responses in IL-6KO mice

**Figure 6:**
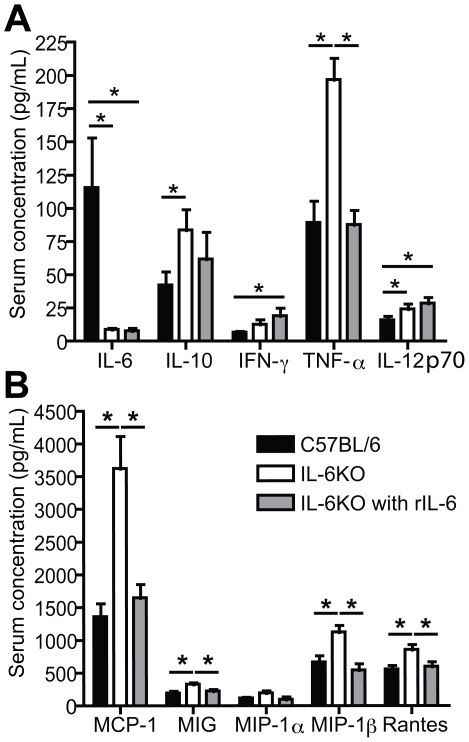
Injection of rIL-6 in IL-6KO mice decreased early inflammatory responses at 3 days post infection. (A) The serum concentrations of the cytokines IL-12p70, IL-6, IL-10, IFNγ and TNF-α were monitored following treatment with CB3/LPS by a BD cytometric bead array inflammation assay at day 3 PI (C57BL/6 with DMEM n = 10, IL-6KO with DMEM n = 9, IL-6KO with rIL-6 n = 10) (black bars indicate wt mice with DMEM, white bars indicate IL-6KO mice with DMEM, grey bars indicate IL-6KO mice with rIL-6) (mean±SE, *p<0.05). TNF-α, IL-10 and IL-12p70 levels were significantly increased in the IL-6KO mice compared to wild type controls. rIL-6 treatment was sufficient to decrease the serum concentration of IL-10 and TNF-α. (B) The serum concentrations of the chemokines MCP-1, MIP-1α, MIP-1β, MIG and RANTES were monitored following treatment with CB3/LPS by a BD cytometric bead array flex set assay at day 3 PI (C57BL/6 n = 10, IL-6KO with DMEM n = 9, IL-6KO with rIL-6 n = 10) (black bars indicate wt mice with DMEM, white bars indicate IL-6KO mice with DMEM, grey bars indicate IL-6KO mice with rIL-6) (mean±SE, *p<0.05). All chemokine levels were increased in the IL-6KO mice compared to wild type controls. Treatment with rIL-6 was sufficient to decrease the levels of all the chemokines.

To characterize the changes following rIL-6 administration, we monitored the cytokine milieu at day 3 PI. IL-6KO mice were observed to have significantly increased serum levels of TNF-α, IL-10, MCP-1, MIP-1β, MIG and RANTES compared to wild type mice at 3 days post CB3/LPS treatment (Figure 6a and 6b). The MIP-1α levels were also increased in the IL-6KO mice however the increase was not significant. IL-6KO mice treated with rIL-6 during the early immune response following CB3/LPS infection had significantly decreased serum levels of TNF-α, IL-10, MCP-1, MIP-1β, MIG and RANTES as well as decreases in MIP-1α (Figure 6a and 6b). This decreased early inflammatory response correlated with decreased chronic disease severity at 28 days post treatment (Figure 5a and 5b) and demonstrates that IL-6 production during the initiation of disease regulates the early immune activation, which in turn determines the severity of chronic disease.

In addition to changes in TNF-α and IL-10, the cytokines IFN-γ and IL-12p70 were differentially expressed between the wild type and knockout mice. IL-12p70 was significantly increased in the IL-6KO mice with and without rIL-6 treatment compared to wild type controls while IFN-γ was increased in the IL-6KO treated with rIL-6 compared to wild type controls. While these increases do not correlate with disease severity, it is possible that they may be a mechanism of disease amelioration following rIL-6 treatment.

### Increased cardiac infiltration in rIL-6 treated IL-6KO mice

**Figure 7:**
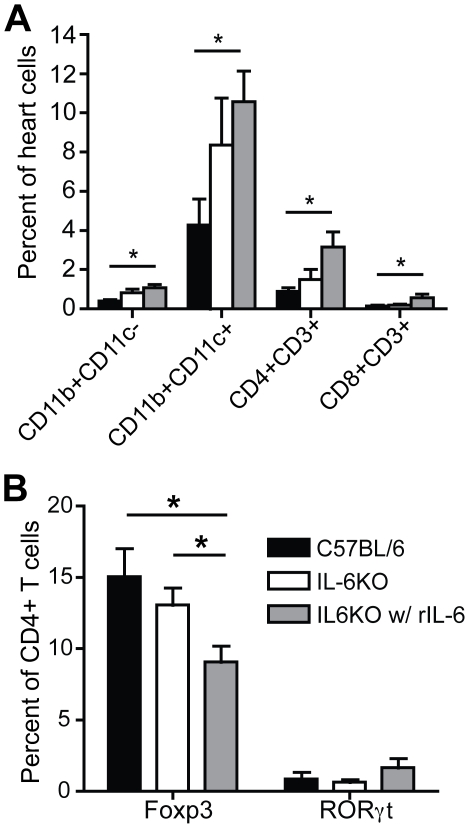
rIL-6 injection in IL-6KO mice increased innate and adaptive immune cell infiltration in the heart. (A) Heart infiltrate was measured by flow cytometry analysis at 10 days post treatment with CB3/LPS with or without rIL-6 treatment. Cardiac infiltrate was stained to determine innate and adaptive immune cell infiltration using the surface markers CD11b, CD11c, CD3, CD4 and CD8. rIL-6 treatment in the IL-6KO mice resulted in increased monocyte/macrophages (CD11b+CD11c− and CD11b+CD11c+), CD4+ T cells (CD3+CD4+) and CD8+ T cells (CD3+CD8+) at 10 days post treatment compared to wt and IL-6KO mice treated with DMEM (C57BL/6 with DMEM n = 14, IL-6KO with DMEM n = 10, IL-6KO with rIL-6 n = 11) (black bars indicate wt mice with DMEM, white bars indicate IL-6KO mice with DMEM, grey bars indicate IL-6KO mice with rIL-6) (mean±SE, *p<0.05). (B) The percent of CD4+ cells that are Treg or Th17 cells was determined by flow cytometry analysis of cardiac infiltrate at 10 days post CB3/LPS treatment. Treg cells were identified by the presence of the surface marker CD4 and the transcription factor Foxp3 while Th17 cells were identified by the presence of the surface marker CD4 and the transcription factor RORγt. rIL-6 treated IL-6KO mice contained a significantly lower percentage of CD4+ cells that were Treg cells compared to the DMEM treated wild type controls and DMEM treated IL-6KO mice. Minimal Th17 cells were observed in the heart regardless of treatment (C57BL/6 with DMEM n = 7, IL-6KO with DMEM n = 9, IL-6KO with rIL-6 n = 8) (black bars indicate wt mice with DMEM, white bars indicate IL-6KO mice with DMEM, grey bars indicate IL-6KO mice with rIL-6) (mean±SE, *p<0.05).

As the chemokine production correlated with disease severity, the immune cell infiltration in the heart was characterized at the peak of the acute stage of disease (day 10 PI) in the wild type and IL-6KO mice with and without rIL-6 treatment. As observed at day 7 PI, IL-6KO mice treated with DMEM had significantly increased CD11b+, CD11C− macrophage/monocyte infiltration compared to wild type controls at day 10 PI. Unexpectedly, while treatment with rIL-6 resulted in decreased chemokine production, increased cardiac infiltration was observed as increases in the percentage of CD11b+CD11c− and CD11b+CD11c+ monocyte/macrophages and CD4+ and CD8+ T cells (Figure 7a, Figure S4a). Specifically, the CD11b+CD11c− monocyte/macrophage population was 72.1%±1.6% F4/80 positive and 74.5%±0.7% CD14 positive, while the CD11b+CD11c+ monocytes/macrophage population was 83.5%±0.8% F4/80 positive and 81.0%±3.2% CD14 positive.

Further, within the cardiac infiltrating CD4+ cell population at day 10 PI, the proportion of Treg cells to CD4+ T cells did not significantly differ between the wt and IL-6KO mice. IL-6KO mice treated with rIL-6 had a significantly smaller proportion of CD4+ Tregs (Figure 7b, Figure S4b) despite a significantly greater number of infiltrating CD4+ T cells. This suggests that Treg suppression is not responsible for the decrease in inflammation and disease observed in rIL-6 treated IL-6KO mice and that as expected, the presence of IL-6 is reducing the proportion of Tregs to effector T cells by day 10 PI. Interestingly, the IL-6 treatment results in a large number of infiltrating lymphocytes, yet this is commensurate with less observed pathology by day 28. Further, addition of rIL-6 decreased the regulation of the CD4+ T cell responses and therefore increases the adaptive immune response within the heart.

Additonally, minimal Th17 cells were found in wt or IL-6KO mice, as measured by expression of the Th17 specific transcription factor RORγt at day 10 PI, regardless of the treatment (Figure 7b, Figure S4b) suggesting that Th17 cells do not play a major pathogenic role in viral-mediated autoimmune myocarditis development.

Collectively, these results suggest that the four day administration of rIL-6 does not completely replicate the response of a wt IL-6 replete mouse and instead is responsible for a change in the make-up of the infiltrating cell population following infection (day 10) that is responsible for the control in the severity of late stage chronic disease.

## Discussion

Herein, we report that following coxsackievirus infection, IL-6KO mice develop increased chronic autoimmune myocarditis. This result is in contrast to previous reports that IL-6 deficient mice were resistant to induction of autoimmune disease including experimental-induced myocarditis [2]. The study of experimental-induced disease lacks the complications of the viral infection and the early immune response that is essential to specifically clear virus from the host and is also responsible for triggering and controlling a pluripotent autoreactive response.

In the absence of IL-6, we observed greater severity of chronic myocarditis that correlated with changes in the early immune response to virus infection. Most of the differences observed were prior to the peak of virus infection in the heart and clearance from the animal. Specifically, we observed an increase in systemic cytokine/chemokine production, splenic T cell activation and monocyte/macrophage infiltration in the heart. This heightened disease severity was not due to an inability to control viral replication as there was no difference in the viral titer in the heart at the peak of infection. Further indicating a regulatory role for IL-6, rIL-6 treatment during the early immune response was sufficient to decrease the early inflammatory response with a corresponding decrease in the chronic disease. These results strongly suggest that IL-6 regulates and controls the initial host response to pathogen insult and that absence of IL-6 early post infection leaves the host susceptible to increased long-term complications of autoimmune myocarditis.

IL-6 has a pathogenic role in experimental autoimmune models such as EAM [2], EAE [11], pristane-induced lupus [12], experimental-induced arthritis [13], [14] and autoimmune myasthenia gravis [15]. Mice deficient in this cytokine were resistant to autoimmune disease development [2], [11], [12], [13], [14], [15]. Our results, however, clearly show induction of chronic disease with heightened severity in the absence of IL-6. Therefore, viral induction of disease follows a different disease pathogenesis with a more complex immune response in which cytokines, such as IL-6, have varied specific roles compared to experimental models of autoimmunity. Our results suggest a regulatory role for IL-6 following viral infection. Previously, the addition of recombinant IL-6 following encephalomyocarditis virus (EMCV) infection resulted in a decrease in disease severity of autoimmune myocarditis [3]. However, EMCV infection in mice which overexpress IL-6 leads to increased disease severity [5]. These results and ours suggest that IL-6 functions in a delicate balance as a regulator. Early after infection, IL-6 acts to dampen the strength of initial anti-viral immune response to reduce the risk of developing autoimmunity, however, its long-term presence as a chronic participant leads to heightened chronic disease.

Specifically, IL-6 is an essential participant in the acute phase response following viral infection. IL-6 deficient mice were unable to control infection by vaccinia virus and lymphocytic choriomeningitis virus (LCMV) [6], [19]. In terms of CB3, an inability to control the viral infection would lead to greater viral and host directed cardiac damage and subsequently increase chronic disease. We observed no differences in the quantity of replicative virus between the hearts of IL-6KO and wt mice post infection. This suggests that the ability of IL-6 deficient hosts to control CB3 replication was not an issue. Our data suggests that for CB3 infection, the increase in disease severity is a consequence of a dysregulated immune response due to lack of IL-6 throughout the process.

Our results suggest that IL-6 functions by regulating the balance of cytokines and chemokines during the early host response to infection. Thus, IL-6 control has both local and systemic implications and greatly differentiates disease outcome. Changes in the cytokine milieu in the early immune response (day 3 PI) following infection have been demonstrated to later influence the development of chronic autoimmunity [20].

IL-10 levels were found to be increased in the IL-6KO mice. As IL-10 is associated with the anti-inflammatory response and T cell suppression [21] this was a surprising observation. Produced primarily by monocytes and macrophages, IL-10 functions to decrease pro-inflammatory cytokine production by APCs [22]. Production of pro-inflammatory cytokines and IL-10 are initiated following the same stimuli in a cell intrinsic manner and this acts to regulate the inflammatory immune response [22]. Therefore, the early upregulation of IL-10 may serve to control the increased inflammatory cytokines produced in the absence of IL-6. The window of IL-10 expression is short and also observed to be upregulated by LPS treatment alone. In experimental models of autoimmunity including EAE and EAM, IL-10 suppresses disease severity [23], [24]. In contrast, it has been suggested that increased IL-10 following CB3 infection in human monocytes leads to a dysregulation of the immune response to the virus and ultimately allows for the progression to the chronic disease [25]. In fact, increased levels of IL-10 in human patients with fulminant myocarditis correlates with increased mortality rates [26]. IL-10 is also increased in mice following CB3 infection and was again suggested to allow for disease progression from the acute stage of disease to the chronic stage of disease [27], [28]. As such, the increased IL-10 may play a role in increased disease severity. These results highlight the complex balance of pro- and anti-inflammatory mechanisms that are regulated by IL-6.

The increases in TNF-α and MCP-1 observed in the infected IL-6 deficient mice represent a heightened inflammatory response. Both TNF-α and MCP-1 have previously been associated with both disease development and severity, and likely contribute to the increased disease pathology. TNF-α has been instrumental in overcoming genetic resistance to CB3-mediated autoimmune myocarditis [29] and likely acts downstream of LPS signaling. TNF-α was sufficient to induce spontaneous autoimmune myocarditis in mice constitutively overexpressing this cytokine in the heart [30]. In patients with enteroviral-induced myocarditis, TNF-α levels are often increased and are associated with increased myocardial necrosis and cellular infiltration in the myocardium [31]. In our model, TNF-α levels were associated with increased disease severity. In wt mice, similar TNF-α levels were attained following treatment with CB3/LPS or LPS alone regardless of disease outcome suggesting that TNF-α levels are not sufficient for disease observed in these mice. In the absence of IL-6, the level of TNF-α increased with CB3/LPS and was associated with heightened disease severity, clearly indicating a role for TNF-α as an important inflammatory co-factor in disease progression. Taken together, TNF-α functions to increase inflammation associated with the induction of autoimmune heart disease and in the absence of IL-6, an imbalance of TNF-α is induced during the viral response resulting in heightened disease pathology.

MCP-1 is a potent chemoattractant for mononuclear cells, T cells and NK cells expressing CCR2, the receptor for MCP-1, on their surface [18], [32]. MCP-1 is secreted by endothelial cells [33], muscle cells [34] and most notably, coxsackievirus infected cardiac myocytes [18]. Further, elevated MCP-1 expression has been correlated with immune cell infiltration in the heart following coxsackievirus infection [18]. MCP-1 is expressed in a dose dependent manner during coxsackievirus infection [18] and increases over the course of infection thus suggesting that it benefits the host response to CB3 infection. Further, mice deficient in MCP-1 or CCR2 are resistant to the development of experimental-induced autoimmune myocarditis [35]. Heart specific expression of MCP-1 is sufficient to induce monocyte/macrophage migration to the heart leading to myocarditis and fibrosis [36]. This suggests that MCP-1 and the migration of monocytes/macrophages to the heart is integral to the developing cardiac lesions.

In our model, the absence of IL-6 results in increased early expression of MCP-1 associated with increased monocyte/macrophage infiltration during both the acute infection and the later chronic stage of disease. In the absence of IL-6, this increase in mononuclear cell migration to the heart could lead to heightened cardiopathology. Increased levels of macrophages and monocytes could lead to greater levels of uncontrolled damage and lesion development without a concomitant increase in viral replication or viral directed pathology. However, the further increase in heart infiltrate following rIL-6 treatment would suggest that this is not the mechanism of increased disease severity. In support, increased macrophage infiltration in the heart was seen with rIL-6 treatment following EMCV infection in correlation with decreased chronic disease pathology [3]. It was suggested that the increased macrophage infiltration could lead to faster clearance of virus and therefore decreased cardiac damage. Following rIL-6 treatment we observed increased innate and adaptive immune cell infiltration in combination with a decreased cytokine inflammatory response. It is likely that this combination results in faster viral clearance and better regulation of the early inflammatory responses and results in decreased chronic disease severity in these rIL-6 treated mice. Since it is likely that both viral and immune directed damage are controlling the outcome, the lack of IL- 6 results in slower clearance with less regulation of the early inflammatory responses leading to more inflammatory activity in the heart and results in heightened chronic disease and cardiac damage.

We provide evidence that IL-6 acts to regulate the early immune response following coxsackievirus infection. Regulation of the early response in turn controls the severity of the subsequent chronic disease pathology. Immune factors associated with the increased disease severity included early T cell activation and inflammatory cytokine and chemokine responses following disease induction. Without IL-6 to regulate the early immune response after infection, the heightened early inflammatory response leads to increased chronic myocarditis severity as the disease progresses. These results suggest that, contrary to what was suggested by results from experimental models of disease, anti-IL-6 treatment following viral-induced myocarditis could result in increased damage to the host.

## Supporting Information

Figure S1 IL-6KO mice have increased expression of the early activation marker CD69 at 3 days PI. (A) Representative flow cytometry analysis of splenocyte T cell activation at 3 days post DMEM, LPS, CB3 or CB3/LPS treatment. Activated T cells were identified by immunostaining to the surface markers CD4, CD8 and CD69. CD69 expression was significantly increased on both CD4+ and CD8+ T lymphocytes at 3 days post infection (DMEM: C57BL/6 n = 4, IL-6KO n = 4) (LPS: C57BL/6 n = 7, IL-6KO n = 8) (CB3: C57BL/6 n = 8, IL-6KO n = 18) (CB3/LPS: C57BL/6 n = 8, IL-6KO n = 17) (grey line indicate wt mice, black line indicate IL-6KO mice, light grey area indicates isotype control). (B) Representative flow cytometry analysis of splenocyte T cell activation at 7 days post DMEM, LPS, CB3 or CB3/LPS treatment. Activated T cells were identified by immunostaining to the surface markers CD4, CD8 and CD69. CD69 expression was not significantly different on both CD4+ and CD8+ T lymphocytes at 7 days post infection (DMEM: C57BL/6 n = 4, IL-6KO n = 4) (LPS: C57BL/6 n = 4, IL-6KO n = 4) (CB3: C57BL/6 n = 4, IL-6KO n = 4) (CB3/LPS: C57BL/6 n = 4, IL-6KO n = 5) (grey line indicate wt mice, black line indicate IL-6KO mice, light grey area indicates isotype control).
(0.20 MB TIF)

Figure S2 IL-6KO mice have decreased regulatory T cells. (A) Representative flow cytometry analysis of splenocyte regulatory T cells 3 days post DMEM, LPS, CB3 or CB3/LPS treatment. Treg cells were identified by immunostaining for expression of CD4 and the transcription factor Foxp3. IL-6KO mice contained a significantly lower percentage of CD4+ Foxp3+ cells compared to the wild type controls with DMEM treatment (DMEM: C57BL/6 n = 8, IL-6KO n = 8). However, following treatment with CB3 or CB3/LPS but not LPS alone, the percentage of CD4+ Foxp3+ cells was not significantly different (LPS: C57BL/6 n = 8, IL-6KO n = 8) (CB3: C57BL/6 n = 8, IL-6KO n = 8) (CB3/LPS: C57BL/6 n = 9, IL-6KO n = 9) (grey line indicate wt mice, black line indicate IL-6KO mice, light grey area indicates isotype control) suggesting sufficient proportions of Treg cells are present to immunosuppress disease. (B) Representative flow cytometry analysis of splenocyte regulatory T cells 7 days post DMEM, LPS, CB3 or CB3/LPS treatment. Treg cells were identified by immunostaining to surface CD4 and the transcription factor Foxp3. IL-6KO mice contained a significantly lower percentage of CD4+ Foxp3+ cells compared to the wild type controls with DMEM treatment (DMEM: C57BL/6 n = 7, IL-6KO n = 7). However, following treatment with CB3 or CB3/LPS but not LPS alone, the percentage of CD4+ Foxp3+ cells was not significantly different (LPS: C57BL/6 n = 12, IL-6KO n = 14) (CB3: C57BL/6 n = 11, IL-6KO n = 17) (CB3/LPS: C57BL/6 n = 13, IL-6KO n = 18) (grey line indicate wt mice, black line indicate IL-6KO mice, light grey area indicates isotype control).
(0.65 MB TIF)

Figure S3 IL-6KO mice have an increased number of monocyte/macrophage cells in the heart at 7 days post infection. Heart infiltrate was measured by flow cytometry analysis at 7 days post treatment with CB3/LPS. Cardiac infiltrate was determined based on forward and side scatter as well as CD11b and CD11c immunostaining. IL-6KO mice had significantly increased monocyte/macrophage infiltration at 7 days post treatment compared to wt mice (C57BL/6 n = 9, IL-6KO n = 11) (black bars indicate wt mice, white bars indicate IL-6KO mice) (mean±SE, *p<0.05).
(0.19 MB TIF)

Figure S4 rIL-6 injection in IL-6KO mice increased the number of innate and adaptive immune cells infiltrating in the heart at 10 days post infection. (A) Heart infiltrate was measured by flow cytometry analysis at 10 days post treatment with CB3/LPS with or without rIL-6 treatment. Cardiac infiltrate was immunostained to determine innate and adaptive immune cell infiltration using the surface markers CD11b, CD11c, CD3, CD4 and CD8. rIL-6 treatment in the IL-6KO mice resulted in increased numbers of monocyte/macrophages (CD11b+CD11c− and CD11b+CD11c+), CD4+ T cells (CD3+CD4+) and CD8+ T cells (CD3+CD8+) at 10 days post treatment compared to wt mice (C57BL/6 with DMEM n = 9, IL-6KO with DMEM n = 8, IL-6KO with rIL-6 n = 7) (black bars indicate wt mice with DMEM, white bars indicate IL-6KO mice with DMEM, grey bars indicate IL-6KO mice with rIL-6) (mean±SE, *p<0.05). (B) The number of Treg and Th17 cells was determined by flow cytometry analysis of cardiac infiltrate at 10 days post CB3/LPS treatment. Treg cells were identified by immunostaining for the presence of the surface marker CD4 and the transcription factor Foxp3 while Th17 cells were identified by immunostaining for the presence of the surface marker CD4 and the transcription factor RORγt. rIL-6 treated IL-6KO mice contained a significantly higher number of Treg cells compared to the DMEM treated wild type controls and DMEM treated IL-6KO mice. Minimal Th17 cells were observed in the heart regardless of treatment (C57BL/6 with DMEM n = 7, IL-6KO with DMEM n = 9, IL-6KO with rIL-6 n = 8) (black bars indicate wt mice with DMEM, white bars indicate IL-6KO mice with DMEM, grey bars indicate IL-6KO mice with rIL-6) (mean±SE, *p<0.05).
(0.25 MB TIF)

## References

[1] Brown CA, O'Connell JB (1995). Myocarditis and idiopathic dilated cardiomyopathy.. Am J Med.

[2] Eriksson U, Kurrer MO, Schmitz N, Marsch SC, Fontana A (2003). Interleukin-6-deficient mice resist development of autoimmune myocarditis associated with impaired upregulation of complement C3.. Circulation.

[3] Kanda T, McManus JE, Nagai R, Imai S, Suzuki T (1996). Modification of viral myocarditis in mice by interleukin-6.. Circ Res.

[4] Kanda T, Takahashi T (2004). Interleukin-6 and cardiovascular diseases.. Jpn Heart J.

[5] Tanaka T, Kanda T, McManus BM, Kanai H, Akiyama H (2001). Overexpression of interleukin-6 aggravates viral myocarditis: impaired increase in tumor necrosis factor-alpha.. J Mol Cell Cardiol.

[6] Kopf M, Ramsay A, Brombacher F, Baumann H, Freer G (1995). Pleiotropic defects of IL-6-deficient mice including early hematopoiesis, T and B cell function, and acute phase responses.. Ann N Y Acad Sci.

[7] Pasare C, Medzhitov R (2003). Toll pathway-dependent blockade of CD4+CD25+ T cell-mediated suppression by dendritic cells.. Science.

[8] Bettelli E, Carrier Y, Gao W, Korn T, Strom TB (2006). Reciprocal developmental pathways for the generation of pathogenic effector TH17 and regulatory T cells.. Nature.

[9] Korn T, Bettelli E, Gao W, Awasthi A, Jager A (2007). IL-21 initiates an alternative pathway to induce proinflammatory T(H)17 cells.. Nature.

[10] Yajima T, Yasukawa H, Jeon ES, Xiong D, Dorner A (2006). Innate defense mechanism against virus infection within the cardiac myocyte requiring gp130-STAT3 signaling.. Circulation.

[11] Samoilova EB, Horton JL, Hilliard B, Liu TS, Chen Y (1998). IL-6-deficient mice are resistant to experimental autoimmune encephalomyelitis: roles of IL-6 in the activation and differentiation of autoreactive T cells.. J Immunol.

[12] Richards HB, Satoh M, Shaw M, Libert C, Poli V (1998). Interleukin 6 dependence of anti-DNA antibody production: evidence for two pathways of autoantibody formation in pristane-induced lupus.. J Exp Med.

[13] Alonzi T, Fattori E, Lazzaro D, Costa P, Probert L (1998). Interleukin 6 is required for the development of collagen-induced arthritis.. J Exp Med.

[14] Ohshima S, Saeki Y, Mima T, Sasai M, Nishioka K (1998). Interleukin 6 plays a key role in the development of antigen-induced arthritis.. Proc Natl Acad Sci U S A.

[15] Deng C, Goluszko E, Tuzun E, Yang H, Christadoss P (2002). Resistance to experimental autoimmune myasthenia gravis in IL-6-deficient mice is associated with reduced germinal center formation and C3 production.. J Immunol.

[16] Horwitz MS, La Cava A, Fine C, Rodriguez E, Ilic A (2000). Pancreatic expression of interferon-gamma protects mice from lethal coxsackievirus B3 infection and subsequent myocarditis.. Nat Med.

[17] Lane JR, Neumann DA, Lafond-Walker A, Herskowitz A, Rose NR (1991). LPS promotes CB3-induced myocarditis in resistant B10.A mice.. Cell Immunol.

[18] Shen Y, Xu W, Chu YW, Wang Y, Liu QS (2004). Coxsackievirus group B type 3 infection upregulates expression of monocyte chemoattractant protein 1 in cardiac myocytes, which leads to enhanced migration of mononuclear cells in viral myocarditis.. J Virol.

[19] Kopf M, Baumann H, Freer G, Freudenberg M, Lamers M (1994). Impaired immune and acute-phase responses in interleukin-6-deficient mice.. Nature.

[20] Richer M, Poffenberger MC, Horwitz MS (2007). Early inflammatory responses direct chronic autoimmunity development in the heart following coxsackievirus infection.. Future Virology.

[21] Blackburn SD, Wherry EJ (2007). IL-10, T cell exhaustion and viral persistence.. Trends Microbiol.

[22] Girndt M, Kohler H (2003). Interleukin-10 (IL-10): an update on its relevance for cardiovascular risk.. Nephrol Dial Transplant.

[23] Bettelli E, Nicholson LB, Kuchroo VK (2003). IL-10, a key effector regulatory cytokine in experimental autoimmune encephalomyelitis.. J Autoimmun.

[24] Watanabe K, Nakazawa M, Fuse K, Hanawa H, Kodama M (2001). Protection against autoimmune myocarditis by gene transfer of interleukin-10 by electroporation.. Circulation.

[25] Hofmann P, Schmidtke M, Stelzner A, Gemsa D (2001). Suppression of proinflammatory cytokines and induction of IL-10 in human monocytes after coxsackievirus B3 infection.. J Med Virol.

[26] Nishii M, Inomata T, Takehana H, Takeuchi I, Nakano H (2004). Serum levels of interleukin-10 on admission as a prognostic predictor of human fulminant myocarditis.. J Am Coll Cardiol.

[27] Gluck B, Schmidtke M, Merkle I, Stelzner A, Gemsa D (2001). Persistent expression of cytokines in the chronic stage of CVB3-induced myocarditis in NMRI mice.. J Mol Cell Cardiol.

[28] Schmidtke M, Gluck B, Merkle I, Hofmann P, Stelzner A (2000). Cytokine profiles in heart, spleen, and thymus during the acute stage of experimental coxsackievirus B3-induced chronic myocarditis.. J Med Virol.

[29] Lane JR, Neumann DA, Lafond-Walker A, Herskowitz A, Rose NR (1992). Interleukin 1 or tumor necrosis factor can promote Coxsackie B3-induced myocarditis in resistant B10.A mice.. J Exp Med.

[30] Kubota T, McTiernan CF, Frye CS, Demetris AJ, Feldman AM (1997). Cardiac-specific overexpression of tumor necrosis factor-alpha causes lethal myocarditis in transgenic mice.. J Card Fail.

[31] Calabrese F, Carturan E, Chimenti C, Pieroni M, Agostini C (2004). Overexpression of tumor necrosis factor (TNF)alpha and TNFalpha receptor I in human viral myocarditis: clinicopathologic correlations.. Mod Pathol.

[32] Zhao HF, Ito T, Gibo J, Kawabe K, Oono T (2005). Anti-monocyte chemoattractant protein 1 gene therapy attenuates experimental chronic pancreatitis induced by dibutyltin dichloride in rats.. Gut.

[33] Rollins BJ, Yoshimura T, Leonard EJ, Pober JS (1990). Cytokine-activated human endothelial cells synthesize and secrete a monocyte chemoattractant, MCP-1/JE.. Am J Pathol.

[34] Rollins BJ (1997). Chemokines.. Blood.

[35] Goser S, Ottl R, Brodner A, Dengler TJ, Torzewski J (2005). Critical role for monocyte chemoattractant protein-1 and macrophage inflammatory protein-1alpha in induction of experimental autoimmune myocarditis and effective anti-monocyte chemoattractant protein-1 gene therapy.. Circulation.

[36] Kolattukudy PE, Quach T, Bergese S, Breckenridge S, Hensley J (1998). Myocarditis induced by targeted expression of the MCP-1 gene in murine cardiac muscle.. Am J Pathol.

