# Supplementary figures and images for: Lack of IL-6 during Coxsackievirus Infection Heightens the Early Immune Response Resulting in Increased Severity of Chronic Autoimmune Myocarditis
Source: PLoS One. 2009 Jul 9;4(7):e6207. doi: 10.1371/journal.pone.0006207 (PMC2703827; doi:10.1371/journal.pone.0006207)

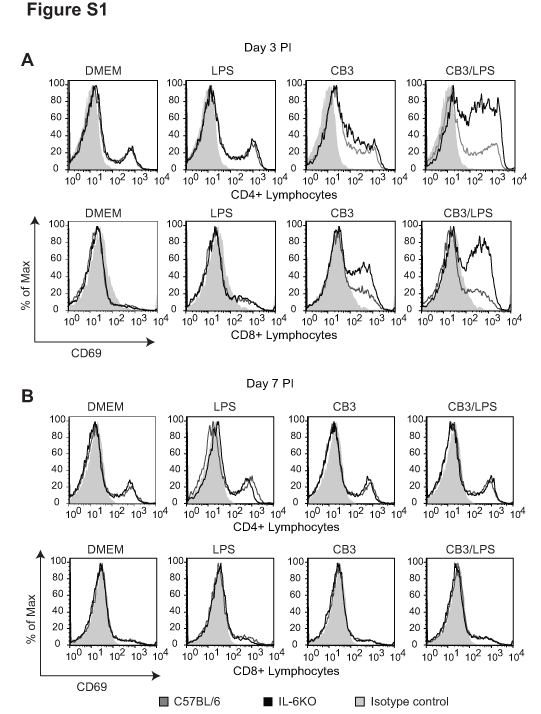

Supplement: Figure S1 — IL-6KO mice have increased expression of the early activation marker CD69 at 3 days PI. (A) Representative flow cytometry analysis of splenocyte T cell activation at 3 days post DMEM, LPS, CB3 or CB3/LPS treatment. Activated T cells were identified by immunostaining to the surface markers CD4, CD8 and CD69. CD69 expression was significantly increased on both CD4+ and CD8+ T lymphocytes at 3 days post infection (DMEM: C57BL/6 n = 4, IL-6KO n = 4) (LPS: C57BL/6 n = 7, IL-6KO n = 8) (CB3: C57BL/6 n = 8, IL-6KO n = 18) (CB3/LPS: C57BL/6 n = 8, IL-6KO n = 17) (grey line indicate wt mice, black line indicate IL-6KO mice, light grey area indicates isotype control). (B) Representative flow cytometry analysis of splenocyte T cell activation at 7 days post DMEM, LPS, CB3 or CB3/LPS treatment. Activated T cells were identified by immunostaining to the surface markers CD4, CD8 and CD69. CD69 expression was not significantly different on both CD4+ and CD8+ T lymphocytes at 7 days post infection (DMEM: C57BL/6 n = 4, IL-6KO n = 4) (LPS: C57BL/6 n = 4, IL-6KO n = 4) (CB3: C57BL/6 n = 4, IL-6KO n = 4) (CB3/LPS: C57BL/6 n = 4, IL-6KO n = 5) (grey line indicate wt mice, black line indicate IL-6KO mice, light grey area indicates isotype control). (0.20 MB TIF) [file pone.0006207.s001.tif]

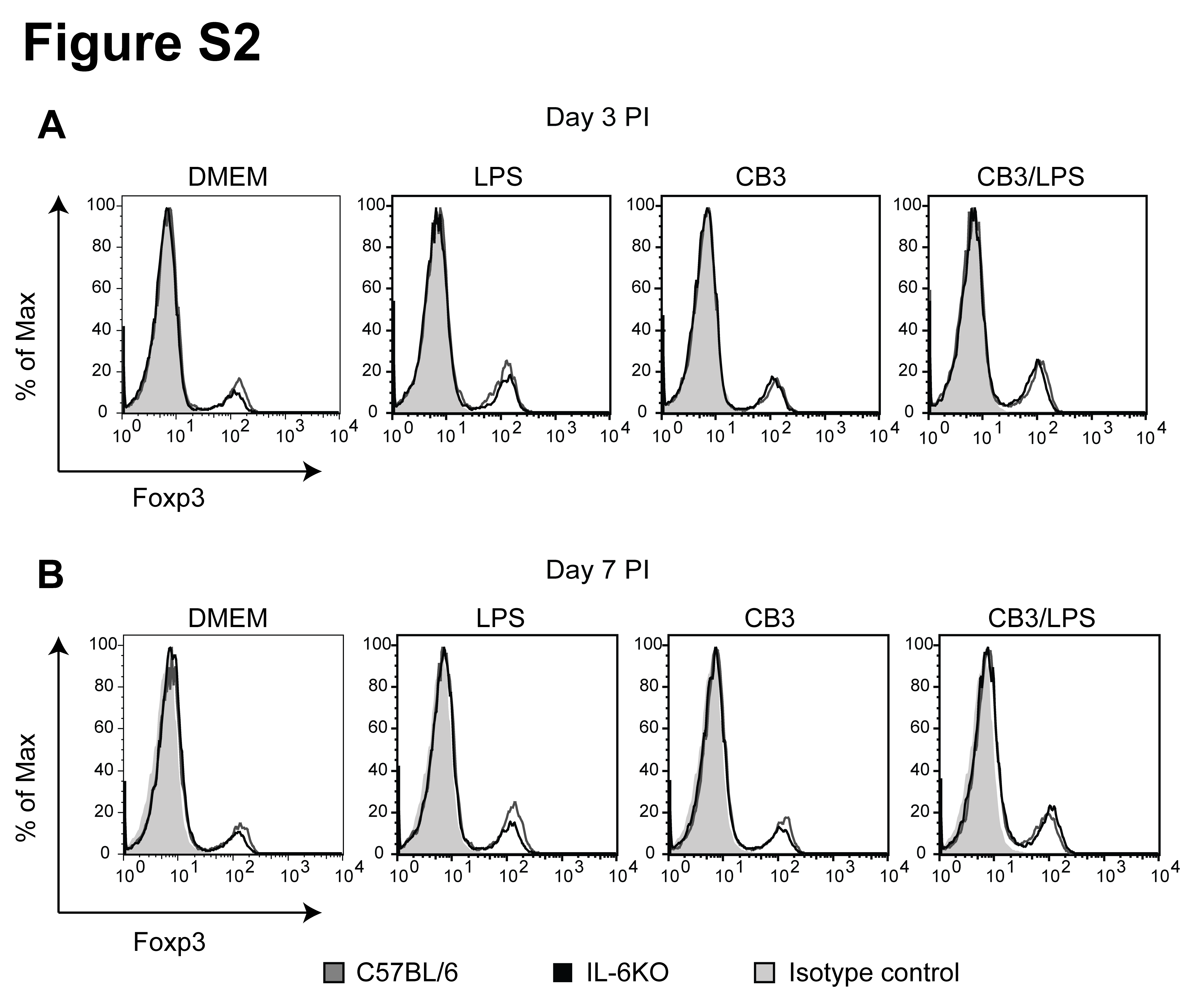

Supplement: Figure S2 — IL-6KO mice have decreased regulatory T cells. (A) Representative flow cytometry analysis of splenocyte regulatory T cells 3 days post DMEM, LPS, CB3 or CB3/LPS treatment. Treg cells were identified by immunostaining for expression of CD4 and the transcription factor Foxp3. IL-6KO mice contained a significantly lower percentage of CD4+ Foxp3+ cells compared to the wild type controls with DMEM treatment (DMEM: C57BL/6 n = 8, IL-6KO n = 8). However, following treatment with CB3 or CB3/LPS but not LPS alone, the percentage of CD4+ Foxp3+ cells was not significantly different (LPS: C57BL/6 n = 8, IL-6KO n = 8) (CB3: C57BL/6 n = 8, IL-6KO n = 8) (CB3/LPS: C57BL/6 n = 9, IL-6KO n = 9) (grey line indicate wt mice, black line indicate IL-6KO mice, light grey area indicates isotype control) suggesting sufficient proportions of Treg cells are present to immunosuppress disease. (B) Representative flow cytometry analysis of splenocyte regulatory T cells 7 days post DMEM, LPS, CB3 or CB3/LPS treatment. Treg cells were identified by immunostaining to surface CD4 and the transcription factor Foxp3. IL-6KO mice contained a significantly lower percentage of CD4+ Foxp3+ cells compared to the wild type controls with DMEM treatment (DMEM: C57BL/6 n = 7, IL-6KO n = 7). However, following treatment with CB3 or CB3/LPS but not LPS alone, the percentage of CD4+ Foxp3+ cells was not significantly different (LPS: C57BL/6 n = 12, IL-6KO n = 14) (CB3: C57BL/6 n = 11, IL-6KO n = 17) (CB3/LPS: C57BL/6 n = 13, IL-6KO n = 18) (grey line indicate wt mice, black line indicate IL-6KO mice, light grey area indicates isotype control). (0.65 MB TIF) [file pone.0006207.s002.tif]

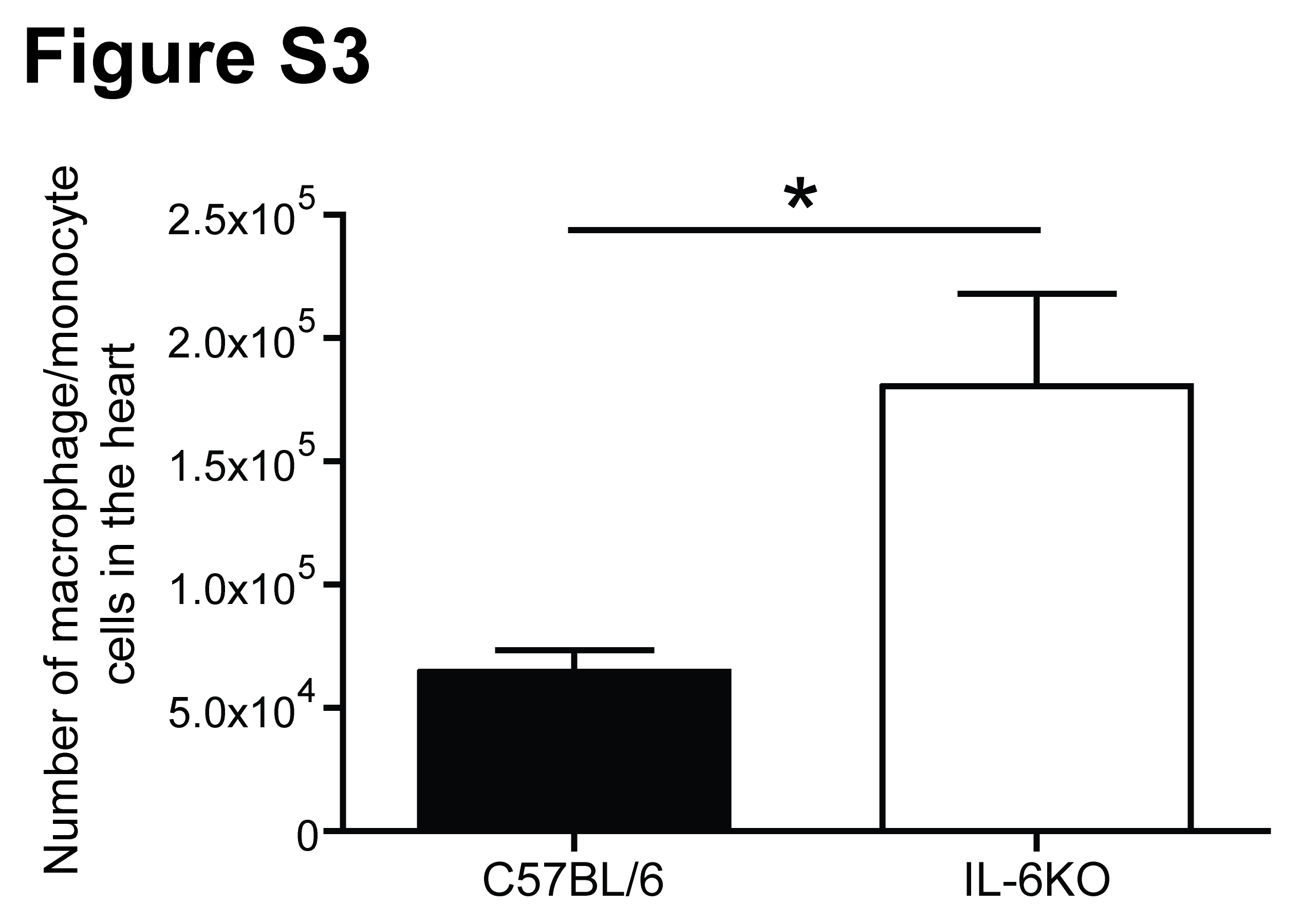

Supplement: Figure S3 — IL-6KO mice have an increased number of monocyte/macrophage cells in the heart at 7 days post infection. Heart infiltrate was measured by flow cytometry analysis at 7 days post treatment with CB3/LPS. Cardiac infiltrate was determined based on forward and side scatter as well as CD11b and CD11c immunostaining. IL-6KO mice had significantly increased monocyte/macrophage infiltration at 7 days post treatment compared to wt mice (C57BL/6 n = 9, IL-6KO n = 11) (black bars indicate wt mice, white bars indicate IL-6KO mice) (mean±SE, *p<0.05). (0.19 MB TIF) [file pone.0006207.s003.tif]

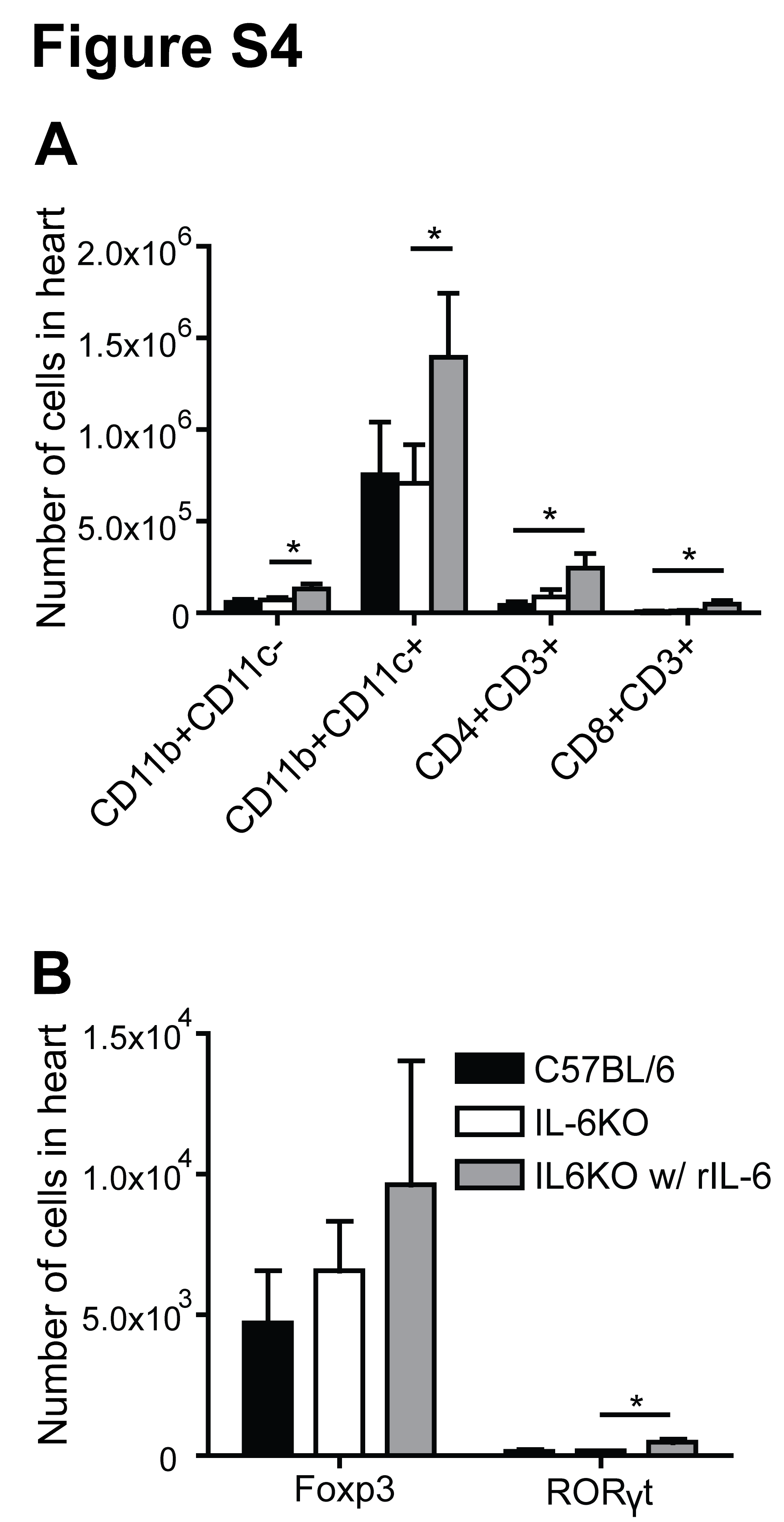

Supplement: Figure S4 — rIL-6 injection in IL-6KO mice increased the number of innate and adaptive immune cells infiltrating in the heart at 10 days post infection. (A) Heart infiltrate was measured by flow cytometry analysis at 10 days post treatment with CB3/LPS with or without rIL-6 treatment. Cardiac infiltrate was immunostained to determine innate and adaptive immune cell infiltration using the surface markers CD11b, CD11c, CD3, CD4 and CD8. rIL-6 treatment in the IL-6KO mice resulted in increased numbers of monocyte/macrophages (CD11b+CD11c− and CD11b+CD11c+), CD4+ T cells (CD3+CD4+) and CD8+ T cells (CD3+CD8+) at 10 days post treatment compared to wt mice (C57BL/6 with DMEM n = 9, IL-6KO with DMEM n = 8, IL-6KO with rIL-6 n = 7) (black bars indicate wt mice with DMEM, white bars indicate IL-6KO mice with DMEM, grey bars indicate IL-6KO mice with rIL-6) (mean±SE, *p<0.05). (B) The number of Treg and Th17 cells was determined by flow cytometry analysis of cardiac infiltrate at 10 days post CB3/LPS treatment. Treg cells were identified by immunostaining for the presence of the surface marker CD4 and the transcription factor Foxp3 while Th17 cells were identified by immunostaining for the presence of the surface marker CD4 and the transcription factor RORγt. rIL-6 treated IL-6KO mice contained a significantly higher number of Treg cells compared to the DMEM treated wild type controls and DMEM treated IL-6KO mice. Minimal Th17 cells were observed in the heart regardless of treatment (C57BL/6 with DMEM n = 7, IL-6KO with DMEM n = 9, IL-6KO with rIL-6 n = 8) (black bars indicate wt mice with DMEM, white bars indicate IL-6KO mice with DMEM, grey bars indicate IL-6KO mice with rIL-6) (mean±SE, *p<0.05). (0.25 MB TIF) [file pone.0006207.s004.tif]
